# The Possible Role of Anti-Neu5Gc as an Obstacle in Xenotransplantation

**DOI:** 10.3389/fimmu.2020.00622

**Published:** 2020-04-15

**Authors:** Alfred Joseph Tector, Mathilde Mosser, Matthew Tector, Jean-Marie Bach

**Affiliations:** ^1^Department of Surgery, University of Alabama at Birmingham School of Medicine, Birmingham, AL, United States; ^2^Immuno-Endocrinology Unit (IECM), USC1383, Oniris, INRA, Nantes, France

**Keywords:** sialic acid, xenotransplantation, anti-Neu5Gc, graft rejection, pig, human disease, animal model

## Abstract

Seventy to ninety percentage of preformed xenoreactive antibodies in human serum bind to the galactose-α(1,3)-galactose Gal epitope, and the creation of Gal knockout (KO) pigs has eliminated hyperacute rejection as a barrier to xenotransplantation. Now other glycan antigens are barriers to move ahead with xenotransplantation, and the N-glycolyl neuraminic acid, Neu5Gc (or Hanganutziu-Deicher antigen), is also a major pig xenoantigen. Humans have anti-Neu5Gc antibodies. Several data indicate a strong immunogenicity of Neu5Gc in humans that may contribute to an important part in antibody-dependent injury to pig xenografts. Pig islets express Neu5Gc, which reacted with diet-derived human antibodies and mice deleted for Neu5Gc reject pancreatic islets from wild-type counterpart. However, Neu5Gc positive heart were not rejected in Neu5Gc KO mice indicating that the role of Neu5Gc-specific antibodies has to be nuanced and depend of the graft situation parameters (organ/tissue, recipient, implication of other glycan antigens). Recently generated Gal/Neu5Gc KO pigs eliminate the expression of Gal and Neu5Gc, and improve the crossmatch of humans with the pig. This review summarizes the current and recent experimental and (pre)clinical data on the Neu5Gc immunogenicity and emphasize of the potential impact of anti-Neu5Gc antibodies in limiting xenotransplantation in humans.

## Introduction

Organ transplantation is the treatment of choice for end-stage organ failure, but there are not enough human donors to transplant everyone who could benefit. In the United States alone, today more than 110,000 patients are on the United Network for Organ Sharing transplant waitlist, and just over 39,000 patients are transplanted each year (1). In Europe, in 2017, more than 144,000 patients are on waiting list, while almost 43,000 patients are transplanted per year (2). Many patients die before an organ becomes available, while others are never put on the list because they are too sick to wait for a donor organ. Xenotransplantation using pig organs could solve this shortage, but progress toward the clinic has been limited because humans possess antibodies to pig cells that trigger rejection of the graft immediately following graft reperfusion. One major obstacle of xenotransplantation is the rejection of the graft, often obtained from pig, the major candidate for xenotransplantation, by preformed and elicited anti-pig antibodies. The development of cloning for pigs coupled with the advances in targeted genome editing have made it possible to create pigs devoid of xenoantigens to which the xenoreactive antibodies bind (3–6).

A newborn human has few xenoreactive antibodies at birth, but by the first few months of age they have developed a mature xenoreactive antibody repertoire (7, 8). The first xenoantigen to be deleted in the pig was the α-1,3-gal (Gal) glycoprotein epitope that was produced by the α-1,3-galactosyltransferase enzyme (GGTA1) in pig cells (9). Since humans (and Old World monkeys) have deleted this gene during the course of evolution, they do not produce Gal (10, 11), and so they produce antibodies against this epitope when they encounter it during bacterial colonization of their gut during infancy (12). It has been suggested that pathogen bacteria, parasites, vector-borne pathogens and heat stable α-Gal-containing proteins in mammalian meat are other source of anti-Gal immunization in humans [reviewed in (13, 14)]. A Gal antigen is also synthesized in the globosphingolipid metabolism by the isoglobotrihexosylceramide 3 synthase (iGb3S, also called alpha-1,3-galactosyltransferase 2 [gene name *A3GALT2*)]. This enzyme is not functional in human (15). Both pig and mouse express the glycolipid form of the Gal epitope, which is less immunogenic and less recognized by human anti-Gal antibodies than the glycoprotein form (16–18).

Roughly 70–90% of the antibodies that humans have against the pig bind to the Gal epitope (19). The creation of the Gal knockout (KO) pig was a real advance for xenotransplantation, eliminating hyperacute rejection and improving kidney transplant survival in the pig-to-non-human primate (NHP) from hours to 6–16 days using clinically available immunosuppression. Immunopathological analysis of the rejected kidneys showed that AMR was still the cause of graft failure, suggesting that there were other xenoantigens that needed to be deleted to overcome the humoral barrier to clinical application (20). Despite the addition of human complement regulatory protein and thromboregulatory protein transgenes into Gal KO pigs, graft failure is nearly always secondary to AMR, suggesting that the humoral barrier will remain problematic until such time that it is eliminated through genetic engineering (21–23). Moreover, pre-existing antibodies present in human serum bind to Gal KO pig cells or tissues (24), confirming that non-Gal antigens have to be considered in the xenorejection (25, 26). The N-glycolyl neuraminic sialic acid (Neu5Gc or Hanganutziu-Deicher antigen) is a major sugar xenoantigen contained in glycoproteins and ganglioside glycolipids. In 2002, Alex Zhu et al. identified, using an hemagglutination array, Neu5Gc, as a non-Gal crucial xenoantigen (27).

## Neu5Gc, a Major Non-Gal Glycan Xenoantigen

During the course of evolution [about 3 million years ago (mya) (28)], humans have lost the expression of functional CMAH, which is responsible for the hydroxylation of Neu5Ac (the Neu5Acetylated form of the neuraminidic acid) to create Neu5Gc. The theory is that absence of the CMAH conveyed protective immunity to a prevailing strain of malaria several million years ago (<3.5 mya), so that now humans do not produce Neu5Gc, rather they have Neu5Ac exclusively (19, 29). Consequently, Neu5Gc is seen as foreign by the human immune system when exposure occurs. Contrary to Old World monkeys, New World ones present also a *CMAH* gene inactivation 30 mya but independently from human beings (30). Thus, contrary to Gal KO graft that is assessable in Old World NHP that do not express the GGTA1 (20, 25, 31–33), and as only the New World monkeys and humans exhibit a lack of CMAH expression, the pig-to-NHP animal model is not conclusive to study the immunogenicity and the deleterious effects of anti-Neu5Gc antibodies on vascularized or cellular xenografts and may not provide a direct translation to the clinic.

Exogenous Neu5Gc is incorporated into cell surface lipooligosaccharides of non-typeable *Haemophilus influenzae* in the nasopharynx of humans. Neu5Gc from food is taken up by non-typeable *Haemophilus influenzae*, and humans form antibodies against Neu5Gc as well as against non-typeable *Haemophilus influenzae* (34). Other bacteria of the microbiota have the capacity to take the Neu5Gc from food and to use it as carbon source (35) but their role in inducing an anti-Neu5Gc humoral response is not known. Other human main natural exposure to Neu5Gc comes from diet directly (mammalian meat, especially processed and industrial forms, milk, cheese …). In this case, small quantities of ingested Neu5Gc seem to be absorbed and deposited in low amounts on human epithelial and endothelial cells (36, 37). After micro-pinocytosis by human cells, Neu5Gc is integrated into various glycans and glycolipids, and is then expressed on the cell surface (37–39).

In the context of xenotransplantation, Neu5Gc is largely detected on non-human mammal epithelial cells and accumulates on endothelial cells (38–41). Although the identification of the Neu5Gc antigen has not been as detailed as in other animal species such as mice, rabbits, sheep and cattle, pig expresses the CMAH and Neu5Gc antigens. In wild-type (WT) pigs, the Neu5Gc/Neu5Ac ratio varies in tissues depending on the CMAH activity intensity, but Neu5Gc is thus present in pig heart, kidney, spleen, lung, cornea and liver (42–45). The antigenicity of NPCCs (46) and adult porcine islets (47–49) was also linked to the expression of Neu5Gc epitopes and the presence of Gal antigen (50). Neu5Gc is also largely detected in erythrocytes (51). Pig leukocytes (mainly lymphocytes) released during the perfusion of vascularized pig organ may contribute to xenograft recipient immunization as these cells exhibit the major Neu5Gc-GM_3_ and -GD_3_ gangliosides together with Gal-terminated compounds (52). Invalidation of the gene encoding for the GGTA1 seems to increase the expression of Neu5Gc gangliosides and antigens (43, 53), and produces a raise and diversification of acidic glycolipid-specific antibodies after transplantation of a Gal KO heart into baboon (43). In this last study, these anti-acidic glycolipid induced antibodies are very probably not specific for Neu5G since the baboons express this antigen like the pig. However, we can suspect that a large anti-acidic xenoantigen response including anti-Neu5Gc will increase in human in a similar situation. Moreover, mutating the *CMAH* gene together with the *GGTA1* gene reduces antibody binding of almost all human serum tested compared to *GGTA1* KO (54, 55). Finally, despite a high concentration of Neu5Ac in the brain of vertebrates, Neu5Gc is detected at very low level in mammal brain, concentrated in endothelial cells but absent in neurons, probably in line with a neuronal down regulation of the expression of the CMAH encoding gene (56).

## Neu5Gc Immunogenicity

### Diet-Derived Anti-Neu5Gc in Humans

At least 80% of humans possessed anti-Neu5Gc antibodies in a similar level of anti-Gal antibodies (27, 57). Following exposure to dietary Neu5Gc, anti-Neu5Gc IgG and IgM develop in infants (34). As anti-Gal (58), anti-Neu5Gc antibodies are of the IgA, IgM and IgG isotypes (59) but with a predominance of IgG for anti-Neu5Gc antibodies (27, 59). Contrary to anti-Gal antibodies that are detected at high level in all individuals, anti-Neu5Gc antibodies are found at variable levels (59, 60) and undergo affinity maturation during life (61). These differences could be in line with the putatively lower antigenicity of Neu5Gc than this of Gal. Indeed, in contrast with anti-Gal antibodies that are produced similarly to humans by *Ggta1*^–/–^ mice (62), even if at lower levels (63), and by *GGTA1*^–/–^ pigs (64–66), anti-Neu5Gc are not detected in *Cmah*^–/–^ mice even feed with Neu5Gc carrying food, and could be induced only by a strong immunization with Neu5Gc-loaded non-typeable *Haemophilus influenza* (34), non-microbial Neu5Gc (67) or Neu5Gc positive xenogeneic cells (68), suggesting that anti-Neu5Gc induced in *Cmah*^–/–^ mice are more related to elicited antibodies than diet-derived ones. Besides, many Neu5Gc epitopes on various glycoproteins and glycolipids (69) are targeted by anti-Neu5Gc antibodies, which was not the case of anti-Gal ones that recognized a dominant Gal (Galα1-3Galα1-(3)4GlcNAc-R) epitope on glycoproteins (70). Also, contrary to Gal glycolipids, Neu5Gc glycolipids are recognized by human xenoreactive antibodies (16, 17).

### High Titers of Anti-Neu5Gc Antibodies Elicited in Humans by Animal-Derived Medical Antibody and Biodevice Exposure

The xenograft survival can depend not only on the presence of diet-derived anti-Neu5Gc antibodies, but also and more particularly on the presence of anti-Neu5Gc antibodies elicited at high titers previously in the same patient by animal-derived therapies prior to xenotransplantation. Indeed, beside bacteria and diet, animal-derived biodevices and immunotherapies expose humans to a larger amount of Neu5Gc and produce high titers of anti-Neu5Gc antibodies. Anti-Neu5Gc antibodies were first identified in patients who had been exposed to animal serum, identifying Neu5Gc as a xenoantigens prior to its evaluation as a barrier to clinical xenotransplantation. Purified animal immunoglobulins express Gal and Neu5Gc in the variable Fab region and linked to Asn297 of their Fc domain (71). Rabbit IgG display Neu5Gc, but no Neu5Ac (57). Anti-Neu5Gc IgM and IgG quantified by ELISA toward a large panel of Neu5Gc epitopes (72) increase significantly with a peak at 1 month post-treatment in the serum of non-immunosuppressed patients treated with rabbit polyclonal ATG (73) (Table 1). However, all these patients do not exhibit anti-Neu5Gc antibodies and anti-Neu5Gc IgG were induced vigorously in about 20% of the patients. Glycan microarray shows that pre-existing anti-Neu5Gc IgG rapidly diversify their repertoire of recognition of Neu5Gc epitopes on glycoproteins and glycolipids including new Neu5Gc epitopes not recognized before rabbit ATG administration (74) (Table 1). In immunosuppressed patients, Neu5Gc on rabbit ATG is also antigenic and elicits a higher and diversified anti-Neu5Gc humoral response within the first year post-kidney allotransplantation (57, 75) (Table 1).

**TABLE 1:** Anti-Neu5Gc antibody in Neu5Gc non-concordant xenosituation.

	Anti-Neu5Gc detection method	Delay post-graft	Anti-Neu5Gc pathogenic potential impact	References
**Animal-to-Human**				
Pig fetal islet–like cell clusters	Glycan array	1 to 12 months	N/A	(101)
Pigskin	ELISA, glycan array	Up to 34 years	N/A	(92)
Equine, Rabbit anti-thymocyte globulin	ELISA, glycan array		Cytokine release syndrome and serum sickness	(57, 73–75, 113, 119)
**Pig-to-Neu5Gc KO mouse**				
Neonate pig islet in the *Cmah*^–/–^ mouse	Flow cytometry	7 days	N/A	(102)
**Rodent-to-Neu5Gc KO mouse**				
Neu5Gc^+^ thymocytes in the *Cmah*^–/–^ mouse	Flow cytometry	1 to 4 weeks	*In vitro* cytotoxicity	(68)

Another potential sources of Neu5Gc can come from treatments with biologicals (including monoclonal antibodies, recombinant virus and proteins) produced in CMAH-expressing cells such as CHO cells or other non-human mammalian cells (76). The Cetuximab humanized murine antibody has been implicated in anti-Gal IgE dependent anaphylaxis in some treated patients (77) and exhibit Neu5Gc epitopes that induce anti-Neu5Gc antibodies in the *Cmah*^–/–^ mouse (78). Neu5Gc glycosylations are also detected in Erythropoietin produced in CHO (79, 80). In addition, Neu5Gc derived from animal components and present in the medium can also be incorporated into glycans present on recombinant products.

Pig (and bovine) prosthetic heart valves that are devitalized and decellularized to be less immunogenic for clinical use can have long-term successful function in humans. However, despite the cleaning by glutaraldehyde of all pig cells in these devices, residual Gal and Neu5Gc antigens are detected by using immunohistochemistry, HPLC and mass spectrometry (81–83). Anti-Gal (84) and anti-Neu5Gc IgG detected by using sialoglycan microarrays are elicited after implantation of these valves in humans (81, 85, 86). In this way, these anti-Neu5Gc antibodies bind to pig and bovine valves and commercial valves (81). Valves from *GGTA1/CMAH* KO pigs have a reduced human IgM and IgG binding compared to WT pig valves (85). Anti-Gal antibodies have been shown to mediate an inflammatory calcification of bioprosthetic heart valves (87, 88) and have been implicated in their deterioration (89, 90). It is possible that anti-Neu5Gc that recognized various epitopes on bioprosthetic heart valves could participate to their deterioration too.

The study of anti-Neu5Gc humoral response in patients treated with engineered porcine skin dressings confirms the high immunogenicity of Neu5Gc in humans (91, 92) (Table 1). Linda Scobie et al. examined the serum of 220 burn patients who received pig skin grafts for dermal coverage and found that, beside a moderate enhancement of anti-Gal antibodies, Neu5Gc glycans appear to be major non-Gal xenoantigens recognized by anti-Neu5Gc IgM and IgG that remained elevated in patient’s serum for years (34 years was the longest follow up) (92). Serum from burn patients did not show significant binding to Neu5Ac glycans but exhibit a large preference to N-linked glycans (Neu5Gcα2-6LacNAc/Lac) and to a lesser extent to O-linked glycans (Neu5Gcα2-3Core1).

All these results confirm that induced anti-Neu5Gc antibody represents a major immune response in xenotransplantation.

## Lessons From Xenografts in Human Patients

In addition to the treatment with animal immunoglobulins and dressings with pig skin, xenografts have been done in humans and represent situations of mismatch for Neu5Gc.

### NHP-to-Human

Early modern attempts at clinical xenotransplantation used non-human primates as donors of kidney, heart and liver. NHP and humans have both lost the capacity to synthetize Gal. NHP graft in humans are then fully concordant for Gal. Since NHP have a functional *CMAH* gene contrary to humans and produce Neu5Gc (93), humans have a positive crossmatch to the NHP (55) and it is possible that anti-Neu5Gc antibodies were contributors to graft loss. A (hyper)acute or chronic antibody mediated rejection associated with a transplant dysfunction was described as the cause of graft failure in some few NHP-to-Human cases (94–99). However, the antibody studies in the human recipients of NHP xenografts are rather scant and data for antibodies specific for Neu5Gc are lacking, making the role of these antibodies somewhat speculative in these NHP-to-Human graft failures. In the NHP kidney-to-Human situation precisely analyzed by Apolline Salama and Jean-Paul Soulillou in a recent review (29), most (19 among 21) of the NHP kidneys grafted in humans are functional for at least 10 days and until 9 months, suggesting that, pre-existing in recipients, anti-Neu5Gc antibodies, if implicated in rejection, have a less detrimental effect on the vasculature of the graft than the anti-Gal antibodies could have had in the Pig-to-Human situation but which could not be directly involved in NHP-to-Human situation.

### Pig Pancreatic Islet Xenotransplantation

In the context of pig islet xenografts in humans, donors and recipients are not concordant for both Gal and Neu5Gc. Gal antigen is weakly expressed by adult porcine pancreatic islets, but exhibits a high level of expression on newborn pig islets (50), justifying the editing of the *GGTA1* gene at least for the use of NPCCs in future clinical trials. The expression of the Neu5Gc antigen is detectable in neonatal and adult pig islets (46, 47, 100). The detection of anti-Neu5Gc antibodies in human serum is subject to specific difficulties including the diet, the molecular diversity of Neu5Gc epitopes, and the individual variability in anti-Neu5Gc isotypes and antigen recognition patterns (29). This could explain why no increase in anti-Neu5Gc antibody levels in humans following transplantation with fetal islet-like cell clusters has been detected when a unique Neu5Gc-GM3 antigen was used in the anti-Neu5Gc array (40). However, when a broad spectrum of glycan antigens was tested using a glycan array covering a lot of Neu5Gc different epitopes, anti-Neu5Gc antibodies could be clearly detected in some patients grafted with fetal islets but in a less extent than anti-Gal antibodies (101) (Table 1). Pig islet Neu5Gc and sialic acid antigens were implicated *in vitro* in complement-dependent injury of islets by human antibodies and contributed clearly to the antigenicity of pig islets (46, 47). In one study (100), the absence of Neu5Gc expression on isolated islet cells obtained from *CMAH* KO pig did not reduce human antibody binding. However, recent results indicated clearly that anti-Neu5Gc antibodies were produced in the humanized *Cmah*^–/–^ mouse model following WT and Gal KO neonatal islet grafts (102) (Table 1).

## Diet-Derived and Elicited Anti-Neu5Gc Antibodies, a Different Concern for the Pig Xenografts in Humans?

Pre-existing antibodies present at transplantation (i.e., antibodies following diet exposure and those previously elicited by another Neu5Gc contribution than xenotransplantation, like animal immunoglobulin treatment, skin or devitalized biodevices) would be expected to trigger, together with anti-Gal antibodies, the rejection of a WT pig xenotransplant. The high titer of anti-Neu5Gc circulating antibodies generated by the xenotransplantation in an individual (probably similar to those elicited by prior medical treatments) is likely (1) to drastically reduce the survival of the graft containing Neu5Gc and (2) to increase the risk in recipient of chronic inflammation and of associated diseases such as carcinomas and atherosclerotic vascular disease.

### Diet-Derived Anti-Neu5Gc Antibodies

Antibodies specific for Neu5Gc are the majority among non-Gal-specific antibodies that could impact xenotransplantation (27, 54, 55, 59, 60, 103). Pre-existing anti-Neu5Gc IgG exhibit cell- and/or complement-dependent cytotoxicity toward human cells *in vitro* (104) and ADCC of Neu5Gc positive cells (105). Human sera exhibiting high-titer of general anti-Neu5Gc (and not those without) have been described to lead to endothelial cell activation, complement deposition, E-selectin expression, increased pro-inflammatory cytokine secretion, and altered monocytes functions (103, 106). Diet-derived antibodies specific for Neu5Gc may contribute to aggravate lesions caused by anti-Gal antibodies following a pig xenograft in humans (29).

In vascularized xenografts, similarly to (106) that used human endothelial cells exhibiting high Neu5Gc concentrations to describe the vascular effect of diet-induced anti-Neu5Gc antibodies, Neu5Gc positive endothelium from WT pig could be targeted immediately by diet-derived anti-Neu5Gc antibodies that could induce the degradation of the xenograft function (103). In line with this data, anti-Neu5Gc antibodies described to be able of ADCC reactions (107) may participated to the immediate lyse of WT pig endothelial cells (108). Anyway, the production of anti-Neu5Gc antibodies induced by the WT xenograft will quickly take over the effects of diet-derived anti-Neu5Gc antibodies. For Neu5Gc KO xenograft, however, we cannot exclude local inflammation of the graft after colonization by the endothelial cells of the human recipient loaded by diet-derived Neu5Gc.

### Elicited Anti-Neu5Gc Antibodies

Evoked anti-Neu5Gc antibodies (Table 1), which persist for a long time and are characterized by a diversification of their recognition repertoire, could mediate biological effects different from pre-existing antibodies. The transcriptome of human endothelial cells exhibiting “physiological” amounts of Neu5Gc is differentially affected by diet-derived and elicited anti-Neu5Gc antibodies (109). Of note, the involvement in xenotransplantation of elicited antibodies specific for Neu5Gc will be probably different depending on the individual as animal products, cells, tissues and organs do not induce anti-Neu5Gc antibodies in all treated humans and these antibodies show an individual-related variable affinity and specificity for the multiple Neu5Gc-containing antigens (74, 75, 92, 101, 110).

*In vitro*, anti-Neu5Gc antibodies from immunized *Cmah*^–/–^ mice are implicated in the complement-mediated lysis and ADCC of Neu5Gc positive cells (68) (Table 1). The loss of Neu5Gc during evolution promoting inflammatory macrophages and phagocytosis (111), can thus impacts xeno-tissue and -organ rich in macrophages such as the lung. “Humanized” *Cmah*^–/–^ mouse model that develops elicited anti-Neu5Gc antibodies following Neu5G-positive thymocyte immunization rejects WT Neu5Gc-positive syngeneic islets (68). Since the only difference between donor and recipient was the absence of the *Cmah* gene in the recipient, anti-Neu5Gc antibodies were participants in early graft failure in the murine recipient, allowing to anticipated that it will be the same in humans as almost all-human IgG that bind non-Gal pig antigens are shown to be anti-Neu5Gc and responsible for determinant injury to Gal KO cells and organs (54).

Surprisingly, Neu5Gc KO mice do not reject a murine Neu5Gc positive heart while a Gal KO mouse rejects a mouse heart expressing Gal, suggesting that, in some situations such as perhaps a vascularized graft, the anti-Neu5Gc may add to the anti-Gal response in the hyperacute rejection but is not sufficient alone (68). Today, further investigations on the role of anti-Neu5Gc antibodies are necessary to allow a better comprehension of their potential deleterious role in xenograft survival. It is not clear whether anti-Neu5Gc antibodies could be always implicated in delayed xenograft rejection neither in NHP that express Neu5Gc, nor in humans because of the difficulty to establish a cause-and-effect relationship.

In human recipients, we can suspect that elicited anti-Neu5Gc antibodies may induce or participated to chronic inflammatory phenomena such as chronic vascular inflammation (xenosialitis) and tumor progression (37), owing to the presence of diet-derived Neu5Gc epitopes on endothelial cells (57, 106) and some epithelia (37, 112), creating thus a condition of *in situ* immune complex disease (57). Even in the presence of immunosuppression, anti-Neu5Gc antibodies are evoked and may have deleterious effects (57, 75) but their presence in (non-immunosuppressed) ATG-treated patients does not seem to produce clinical sign of vascular injury (113). As the patterns of glycosylation differ between normal and tumor cells, Neu5Gc incorporated into tumor cells will be perceived as foreign neo-antigens by humans (114, 115), however, their contribution is discussed and appears complex (115, 116). Anti-Neu5Gc induced by animal biodevices and/or xenotransplants may also activate the recipient endothelium and provoke chronic lesions of the recipient own vasculature in several organs and tissues following binding of anti-Neu5Gc on endothelial cells and complement activation (106). Compared to sera without anti-Neu5Gc, human sera containing high titer of ATG-elicited anti-Neu5Gc antibodies induce an increase in transcripts (qRT-PCR) encoding for IL-1β, IL-6, and IL-8 pro-inflammatory cytokines in human endothelial cells (57). However, elicited anti-Neu5Gc antibodies do not seem to induce an inflammatory transcriptomic profile in human endothelial cells loaded with a “physiological” Neu5Gc concentration similar to this obtained from diet (109). It has been also described that Neu5Gc and anti-Neu5Gc antibodies may contribute to exacerbate atherosclerotic cardiovascular disease mediated by xenosialitis (37, 106, 117), infectious mononucleosis (118), early serum sickness disease (57, 113, 119), and autoimmunity (120). This last acceptation may depend of the autoimmune disease considered, as multiple sclerosis seems to be more associated to the decrease of anti-Gal antibodies than to an altered titer in anti-Neu5Gc (121). The repertoire shift of elicited anti-Neu5Gc antibodies and their recognition with higher affinities of new epitopes and new Neu5Gc-containing glycans, may be more related to their deleterious implications than their global titer increase (122) in local inflammation or serum sickness disease.

## Eliminating Neu5Gc as a Problem in Xenotransplantation

New nuclease-based genome editing tools, especially CRISPR/Cas9 have made it very straightforward to delete genes in pig cells to provide a direct evaluation of candidate xenoantigen (5, 123–125). Genes thought to produce xenoantigens can be deleted in an immortalized pig cell line and screened for the presence or absence of xenoreactive antibody binding (126). Neu5Gc was the first such xenoantigen to be tested and validated as a xenoantigens using the nuclease-based genome editing approach (54, 55).

Today, Gal KO pig hearts expressing human CD46 and thrombomodulin exhibit with success a more than 6 months orthotopic function in baboons (127). Immunofluorescence staining of the myocardium and plasma levels of non-Gal xenoantibodies does not indicate humoral rejection of the graft. The translation to the Pig-to-Human clinic situation may probably require the KO of Neu5Gc in donor pig, as the expression of CD46 may be insufficient even if it regulates complement molecules such as c3b and c4b that could be activated by the anti-Neu5Gc response (60).

**FIGURE 1:**
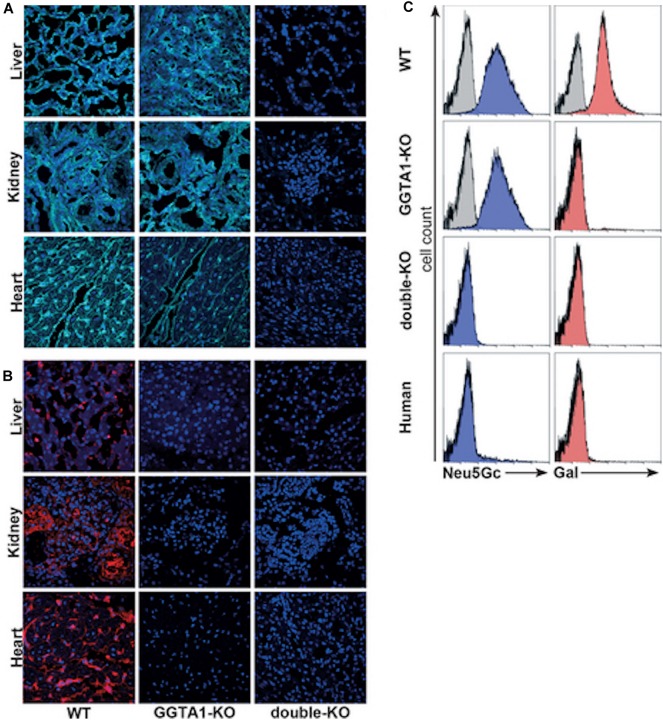
Analysis of carbohydrate epitopes in genetically modified pigs (54). Confocal microscopy of 2-month-old WT, 8-month-old *GGTA1* KO, and 2-day-old *GGTA1/CMAH* double-KO porcine tissues stained with **(A)** anti-Neu5Gc chicken IgY (cyan, Sialix, Vista, CA, United States) and **(B)** IB4 lectin (red). **(C)** Flow cytometric analysis of PBMCs labeled with anti-Neu5Gc antibody (blue) and IB4 lectin (red). Unstained PBMCs were the negative controls for IB4 lectin, and an isotype negative control was used in the anti-Neu5Gc staining. Although shown, the negative controls are difficult to see in some panels because of overlap with the experimental group.

**FIGURE 2:**
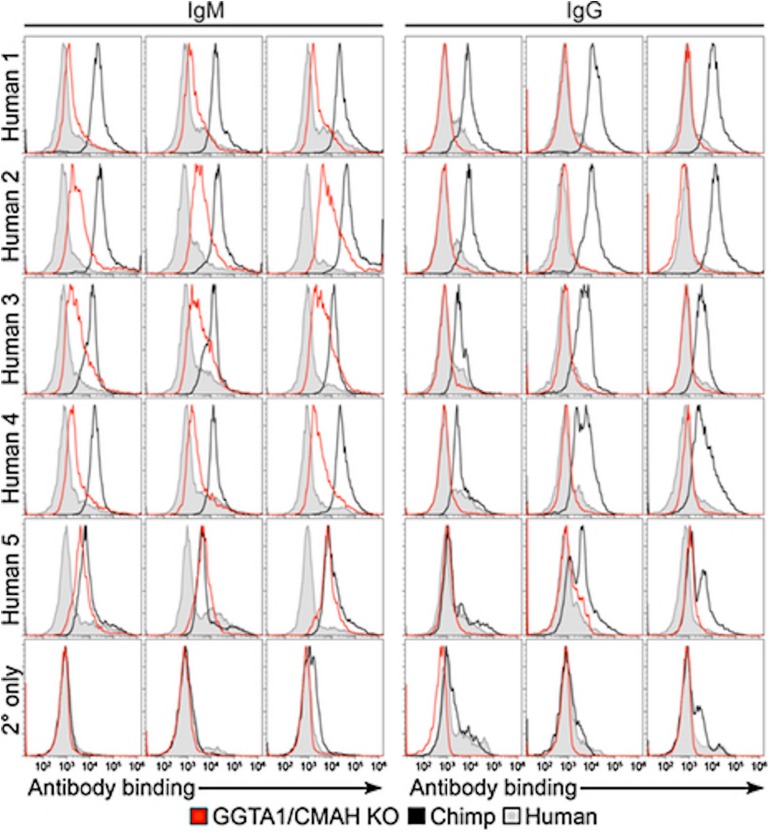
Comparison of human antibody binding to human, porcine or chimpanzee cells (55). PBMCs isolated from humans, chimpanzees and *GGTA1/CMAH* KO pigs incubated with 25% serum collected from five humans. Levels of IgM or IgG binding were detected using fluorescently labeled anti-human IgM or IgG antibodies followed by flow cytometry analysis. Histogram profiles of human IgM and IgG antibody binding are shown for three humans (filled gray), three chimpanzees (black) and three *GGTA1/CMAH* KO pigs (red). Histogram profiles of PBMCs incubated with fluorescently labeled anti-human IgM or IgG antibodies in the absence of human serum are shown to indicate background fluorescence (2° only). CMAH, cytidine monophosphate-N-acetylneuraminic acid hydroxylase; GGTA1, galactosyltransferase.

Zing Finger and then Tale Nucleases were used to perform the first series of multiplex edits in pigs by deleting both GGTA1 and CMAH before using somatic cell nuclear transfer to create new pigs (54, 102, 128–130). These pigs were important for progress in xenotransplantation, and particularly in the evaluation of Neu5Gc as a barrier to clinical implementation. The first finding that was important for xenotransplantation is that multiple engineering could be performed in pigs without compromising viability. This means that it is possible to make multiple modifications in a given pig so that it now takes 6 months to create a pig with many targeted gene edits using nuclease-based technology, as compared to 3 years to create a single homozygous edit to a single gene using homologous recombination and breeding (54, 55). Now with the development of CRISPR/Cas, multiplex engineering has become even simpler (5, 125, 131). The second finding that was just as critical was that the deletion of CMAH eliminated Neu5Gc in all pig tissues. Neu5Gc was expressed in all commonly transplanted tissues; heart, kidney, liver, pancreas, and lung (Figure 1). These pigs made it simple to definitively answer the question of whether Neu5Gc was a significant xenoantigen. Crossmatch results performed using human serum and *GGTA1/CMAH* KO pig cells in flow cytometric crossmatching assays demonstrated that the deletion of Neu5Gc reduced the serum content of IgG and IgM that bound to pig cells significantly. The reduction was significant enough that the crossmatch showed that the binding of xenoreactive antibodies to PBMCs was lower in the *GGTA1/CMAH* KO pigs than it was to chimpanzees (55) (Figure 2).

The immediate question is whether the additional reduction in xenoreactive antibody binding afforded by deleting CMAH on the *GGTA1* KO background has reduced the humoral barrier to the point where clinical implementation is appropriate. Evaluation of the crossmatches from patient serum with the *GGTA1/CMAH* KO pig PBMCs showed that xenoreactive antibody binding, while greatly reduced, was still significant enough that further xenoantigen reduction would be necessary (54, 55). The cellular dependent cytotoxic crossmatches were still positive, indicating that some degree of early AMR would be expected in the absence of any pre-transplant immunomodulatory therapy. Recently, the immunodominant glycan of the Sda antigen group (β1-4-linked GalNAc) has been identified as a significant xenoantigen in humans as well as Old World monkeys (132, 133). The *B4GalNT2* gene encodes for the β1,4 N-acetylgalactosaminyltransferase that catalyzes the terminal addition of GalNAc to a sialic acid modified Gal, producing thus the Sda antigens [reviewed in (134)]. Considering that the *B4GalNT2* gene remains functional in all humans excepted only in about 4% to 5% of humans that are Sda-negative, and that only about half of these Sda-negative individuals exhibit anti-human Sda antibodies especially of the IgM isotype (135–137), it was first predicted that GalNAc would not be an important xenoantigen in the Pig to-Human combination. Once *GGTA1/CMAH/B4GalNT2* KO pigs were created, it was clear that the Sda antigen was a xenoantigen. *GGTA1/CMAH/B4GalNT2* triple KO in pig reduce human antibody binding to pig PBMCs in a flow cytometry crossmatch to clinically acceptable transplant levels (MFI > 5000) in 80% of waitlist patients, 59% of waitlist patients have a complete negative crossmatch to IgG (MFI > 2000), and 31% of patients have a complete negative crossmatch to both IgG and IgM (MFI > 2000) (138). Accurate identification of donor specific pre-existing antibodies in recipient serum is critical to achieve the best post-transplant outcomes. Human IgM and IgG binding to Gal/Neu5Gc/GalNAc KO kidney endothelial pig cells was also significantly reduced compared to cells from Gal and Neu5Gc-deficient pig cells (124, 126). Immunofluorescent staining of Gal/Neu5Gc/GalNAc KO tissues incubated with human serum confirmed a decrease of human IgG and IgM binding in heart, lung and kidney tissues compared to WT pigs contrary to liver, spleen and pancreas of triple KO pigs that linked comparably human immunoglobulins than WT (42). However, in this semi-quantitative study, no comparison with Gal and Neu5Gc KO pig tissue was provided. Finally, anti-Sda IgM and IgG are elicited following blood transfusion of Sda-positive human erythrocytes (139) and may interfere with xenotransplantation.

## Conclusion and Prospects

Naturally occurring anti-Neu5Gc are present in nearly all humans, and creation of Gal/Neu5Gc KO pigs has reduced human antibody binding to the point where the pig has a better crossmatch (fewer antibodies) than the Human-to-Chimpanzee combination. Anti-Neu5Gc (i.e., pre-existing diet-derived antibodies present at transplantation and those elicited by other Neu5Gc contribution than xenotransplantation like animal immunoglobulin treatment, skin or devitalized biodevices) may participate, together with anti-Gal antibodies, to the humoral rejection of the WT pig xenotransplant. Anti-Neu5Gc antibodies elicited by a WT pig graft contribute to the AMR of the graft and may aggravate inflammatory processes in the human recipient caused by the presence of diet-derived Neu5Gc on human cells.

Anti-Neu5Gc antibodies elicited by challenges with xenogeneic tissues or animal-derived immunoglobulins are able to reject pancreas islets in an experimental setting in the mouse. Elicited responses to newly recognized Neu5Gc antigens could be higher detrimental than the diet-derived anti-Neu5Gc responses, suggesting that xenografts lacking Neu5Gc would be safer for the transplant and for the human recipient. The consequences of long-standing exposure to high levels of elicited anti-Neu5Gc antibodies are not well documented and need today to be evaluated. Pig lines have been developed KO for the expression of Gal and Neu5Gc and now also including the KO for GalNAc, in order to eliminate the humoral barrier to clinical xenotransplantation for a great number of people.

## Author Contributions

All authors listed have made a substantial, direct and intellectual contribution to the work, and approved it for publication.

## Conflict of Interest

AT is the founder of Makana Therapeutics and MT now works for that company. J-MB is a co-founder of the Xenothera start-up. The remaining author declares that the research was conducted in the absence of any commercial or financial relationships that could be construed as a potential conflict of interest.

## Abbreviations

ADCC: Ab-dependent cell-mediated cytotoxicity
AMR: antibody-mediated rejection
ATG: anti-thymocyte globulin
CMAH: cytidine monophosphate N-acetylneuraminic acid hydroxylase
DSA: donor-specific antibodies
Gal: galactose-α(1,3)-galactose
GGTA1: α-1,3-galactosyltransferase enzyme
KO: knockout
mya: million years ago
Neu5Gc: N-glycolyl neuraminic acid
NHP: non-human-primate
NPCCs: Neonate pig pancreatic islets or islet-like cell clusters
PBMCs: peripheral blood mononuclear cells
WT: wild-type.
